# Intracranial hemorrhage following drainage of chronic subdural effusion and hematoma: A case report and review of the literature

**DOI:** 10.1002/ibra.12022

**Published:** 2022-02-16

**Authors:** Peng Wen, Wen‐Long Xu, Huan Chen

**Affiliations:** ^1^ Department of Neurosurgery Xuanwu Hospital Capital Medical University Beijing China; ^2^ Department of Neurosurgery The First People's Hospital of Zunyi Zunyi Guizhou China; ^3^ Clinical Pharmacy Department The First People's Hospital of Zunyi Zunyi Guizhou China

**Keywords:** acute intracerebral hemorrhage, chronic subdural hematoma, drainage

## Abstract

Acute intracranial hemorrhage (AIH) after drainage of chronic subdural hematoma is a rare but serious complication. An 86‐year‐old man with bilateral frontotemporal subdural effusion, hematoma, and cerebral hernia was admitted to our department and treated with bilateral burr hole surgery and closed‐system drainage under local anesthesia. After the operation, computed tomography (CT) showed AIH in the left temporal and occipital lobe, and then a series of head CT showed that the hematoma gradually increased day by day. This patient had a medical history of hypertension, diabetes, atrial fibrillation, and taking warfarin. He was treated conservatively, but had not recovered at discharge after 1 month. We reviewed the relevant literature and analyzed the operation opportunity, causes of cerebral hemorrhage, and preventive measures in similar patients. We suppose that the coagulation abnormality and rapid fluctuations of intracranial pressure were the main causes of development of AIH in our patient. Several possible reasons such as brain shift and impaired vascular autoregulation are also associated with postoperative AIH. We must be aware of this complication and keep some preventive measures in our mind.

## Graphical abstract

Patients with chronic subdural effusion may develop chronic subdural hematoma if they do not pay attention to prevention. Similarly, the current patient developed chronic subdural hematoma (A,B). He received an emergency surgery (bilateral skull drilling and drainage), which resulted in acute intracranial hemorrhage after the operation (C). Slow and volume‐limited blood drainage, and recovery of international normalized ratio may be helpful to avoid the complication.

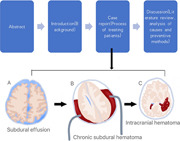

## 1 INTRODUCTION

Chronic subdural hematoma (CSDH) is a common neurosurgical disease, and the incidence rate in the general population is about 1.0–13.1/100,000 per year,^1^ while that in the elderly is about 17–58/100,000 per year.^2^, ^3^ The mortality rate of CSDH is about 0.5%–4%. Currently, burr hole surgery (BHS) with subdural or subgaleal drainage, percutaneous YL‐1 needle drainage, and craniotomy endoscopic treatment have been proven to be effective for CSDH.^4^ Of these, BHS with drainage is the most common surgical procedure, which is minimally invasive, simple, and efficient. Moreover, middle meningeal artery embolization, oral steroids, and atorvastatin can promote the absorption of subdural hematoma or effusion. It has been reported that acute intracranial hemorrhage (AIH) after CSDH drainage is a rare but serious complication.^5^ Most of the reports mainly discussed the reasons for development of AIH in different regions of the brain. However, the causes remain unclear. This paper reports a case involving a patient with subdural effusion, CSDH, and cerebral hernia. The patient developed AIH after receiving BHS. The volume of AIH is associated with changes in international normalized ratios (INR). We reviewed the relevant literature and analyzed the timing of surgery and the causes of bleeding for similar patients.

## 2 CASE REPORT

**Figure 1:**
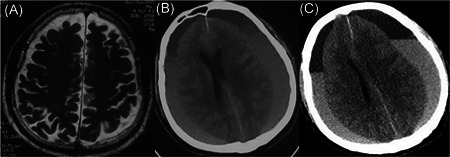
Preoperative head imaging. (A) Before admission, previous magnetic resonance imaging showed bilateral chronic subdural effusion; (B) on admission, computed tomography (CT) showed bilateral chronic subdural hematoma with effusion; (C) 1 day after admission, CT showed that subdural hematoma had increased

**Figure 2:**
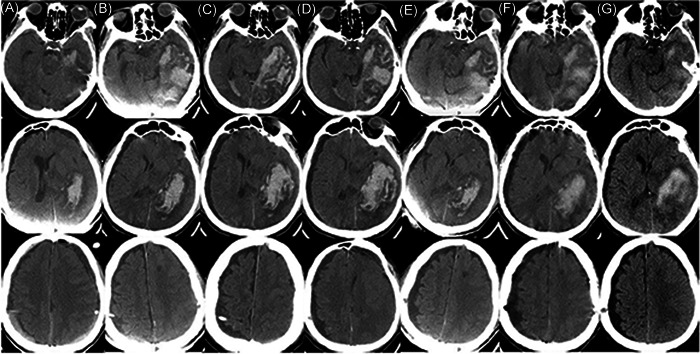
Postoperative brain computed tomography (CT) showed hemorrhage in the temporal lobe, occipital lobe, and the occipital angle of the right lateral ventricle. Columns (A–G) represent cranial CT immediately after the operation and on days 2, 3, 4, 5, 11, and 19 after the operation, respectively

**Figure 3:**
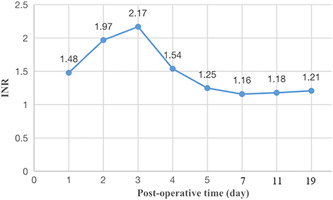
Trend of INR over time. INR, international standardization ratio [Color figure can be viewed at wileyonlinelibrary.com]

An 86‐year‐old man, with a medical history of hypertension, diabetes, and atrial fibrillation, was referred to our hospital with a 2‐month history of memory loss. He had been taking oral warfarin and antihypertensive drugs for a long time, without a history of treatment with antiplatelet drugs. Previous brain computed tomography (CT) (MRI) revealed bilateral frontotemporal subdural effusion (Figure 1A). As there was no obvious neurological symptom, the patient did not receive any treatment. During this visit, the patient suffered a sudden fall and cardiac arrest, and then recovered after artificial cardiopulmonary resuscitation (CPR). He was transferred to the Department of Cardiology and the doctors are going to install a pacemaker for him. An immediate head CT depicted chronic subdural effusion and hematoma in the bilateral hemisphere (Figure 1B). Considering that the patient showed smooth recovery, no obvious neurological deficit was discovered, and the fact that the patient was taking warfarin, a neurosurgeon suggested that watchful waiting was better and coagulation dysfunction should be corrected first for the patient. However, on the second day, the patient's condition worsened, accompanied by disturbance of consciousness, a glasgow coma scale (GCS) score of five points, bilateral pupil diameter of about 4 mm, and slow light reflex. A CT scan showed that subdural hematoma had increased (Figure 1C), the left lateral ventricle was compressed, and the midline had shifted to the right. After getting the consent of the family members, we performed bilateral BHS immediately for him under local anesthesia. First, the operation was performed on the left side with more severe compression, and then on the right side. The closed drainage system was placed under the dura mater at the bilateral parietal tubercle and fixed properly, and the black‐brown bloody liquid flowed out. To save time of the operation, we only performed drainage without using normal saline to irrigate. The drainage volume was about 160 ml from the left hematoma cavity and about 100 ml from the right hematoma cavity. It took about 30 min for the whole operation to be completed, and the blood pressure of the patient was stable during the surgery. Postoperative consciousness and pupil of the patient did not return to normal. A brain CT scan was performed immediately, which indicated AIH in  the left temporal occipital lobe (Figure 2A). The patient was sent to the neuro intensive care unit for drug treatment, and underwent cranial CT examination on the 2nd (Figure 2B), 3rd (Figure 2C), 4th (Figure 2D), 5th (Figure 2E), 11th (Figure 2F), and 19th (Figure 2G) days after the operation. The corresponding INRs are shown (Table 1, Figure 3). One month later, the patient was in stable condition with a GCS score of 7, and was transferred to another hospital for further rehabilitation treatment.

**Table 1:** INR values over time

Postoperative time	INR	Normal limits
Day 1	1.48	0.8–1.2
Day 2	1.97	0.8–1.2
Day 3	2.17	0.8–1.2
Day 4	1.54	0.8–1.2
Day 5	1.25	0.8–1.2
Day 7	1.16	0.8–1.2
Day 11	1.18	0.8–1.2
Day 19	1.21	0.8–1.2

Abbreviation: INR, international standardization ratio.

## 3 DISCUSSION

In the past, most studies showed that the prognosis of CSDH was generally good, but some scholars believe that the trend of the poor prognosis of CSDH is gradually increasing, which is mainly due to the increasing proportion of elderly patients, who tend to have more underlying diseases, higher incidence of postoperative complications, and mortality. The patients with CSDH are often treated when the neurological symptoms occur. There are various treatments for CSDH. The BHS drainage is a simple, minimally invasive, and effective operation. However, complications such as intracranial hemorrhage, infection, epilepsy, pneumocephalus, and hydrocephalus may still occur after the surgery.^6^ Postoperative AIH is a rare and serious complication. Previous reports have shown that hemorrhage can occur in brain parenchyma, cerebellum, brain stem, ventricles of the brain, subarachnoid, or multiple sites of the brain after drainage. This paper reports a case involving a patient with subdural effusion and CSDH who was treated with BHS. Before the operation, he suffered a sudden cardiac arrest and underwent CPR. After the operation, AIH occurred. We discussed the operation method, timing of surgery, the causes of bleeding, and how to avoid postoperative bleeding.

The incidences of CSDH, postoperative complications, and mortality are higher in elderly patients. Karibe et al. reported that the incidence in people older than 65 years of age was 80.1–127.1/10 million, and the peak was concentrated in those older than 80 years of age.^7^ Among the elderly patients, around half may be taking warfarin.^8^ The incidence of CSDH for patients on anticoagulant or antiplatelet drugs was significantly higher than those not taking these drugs.^9^ Consistent with the above study, the present study reports on an 86‐year‐old man with a history of chronic subdural effusion and of taking oral warfarin. CSDH was detected after he came to the hospital. We searched all the similar English reports. Most of the patients were over 60 years old. The proportion of patients over 80 years old was higher. The oldest patient was 88 years old, and the youngest patient was 40 years old. Among the 33 patients reported (Table 2), 11 patients did not indicate the use of coagulation and antiplatelet drugs, and 6 patients had coagulation abnormalities or used anticoagulant and antiplatelet drugs. Ten patients died of various complications, including 4 patients with postoperative brain stem hemorrhage. Only 15 patients recovered well. Despite a series of active treatments, our patient was still in a coma 1 month after surgery.

**Table 2:** A series of related intracranial hemorrhage cases

	Age sex	CSDH side	Clotting profiles	Operation	Drainage amount (ml)	Irrigation	Blood pressure	Interval between drainage and bleeding	Cerebral hemorrhage region
Corrivetti et al.^10^	64 M	Bilateral	Normal	Bilateral parietal burr hole	100	No	Hypertension	1 day	SAH, bilateral
Miyazaki et al.^11^	56 M	Left	Abnormal	Left parietal burr hole	Not reported	No	Well controlled	Immediately	SAH, contralateral
Rusconi et al.^12^	62 M	Right	Normal	Right burr hole	650	No	Hypertension	A few hours	SAH, bilateral
Rusconi et al.^12^	85 M	Right	Not reported	Right burr hole	500	No	Hypertension	1 day	Right SAH, contralateral occipital hematoma
Rusconi et al.^12^	80 M	Right	Not reported	Right burr hole	600	No	Hypertension	1 day	Contralateral temporal hematoma
Zavatto et al.^13^	65 F	Right	Normal	Right burr hole	Not reported	Yes	Normal	1 day	Brain stem hemorrhage
Krishnan et al.^14^	72 M	Right	Normal	Right burr hole	Not reported	No	Normal	4 h	Both cerebellar hemispheres, etc.
Kim et al.^15^	62 M	Right	First time: normal Second: abnormal	Right burr hole	Not reported	No	Not reported	First time: 3 days Second time: 2 days	Subdural Basal ganglia
Park et al.^16^	84 M	Right	Normal	Right burr hole	30	No	Normal	1 h	Subdural
Chang et al.^17^	53 F	Bilateral	Normal	Bilateral burr hole	Right 100 Left 120	Yes	Normal	6 h	Left cerebellar
Vogels et al.^18^	49 F	Bilateral	Normal	Bilateral burr hole	40	Yes	Normal	6 h	Right cerebellar
Vogels et al.^18^	73 M	Right	Abnormal Correct it	Right burr hole	40	Yes	Normal	84 h	Cerebellar vermis
Park et al.^19^	76 M	Bilateral	Normal	Bilateral burr hole	Not reported	No	Normal	2 days	Brain stem
Wang Get al.^20^	88 FM	Right	Normal	Right burr hole	400	Yes	Normal	2 h	SAH SDH
Gurbuz et al.^21^	80 M	Left	Normal	Two burr hole	300	No	Well controlled	2 h	Cerebellar SAH
Rojas‐Medina et al.^22^	58 M	Bilateral	Low‐dose aspirin and clopidogrel Normal	Bilateral burr hole Mini‐craniotomy	300	Yes	Hypertension	First time: few minutes Second: 3 h	SDH Brain stem
Kollatos et al.^23^	43 M	Bilateral	Normal	Bilateral burr hole	300 (4 days)	No	Normal	1 h	SAH cerebellar
Hur et al.^24^	86 M	Bilateral	Not reported	Bilateral burr hole	Right 110 Left 300	No	Normal	4 days	Left cerebellar
Hur et al.^24^	75 M	Bilateral	Not reported	Bilateral burr hole	Right 1440 Left 160	No	Normal	5 days	Right cerebellar
Koller et al.^25^	59 M	Bilateral (effusion)	Normal	Bilateral burr hole	1150 (3 days)	No	Hypertension	4 days	Left cerebellar
Koller et al.^25^	72 M	Bilateral (effusion)	Normal	Bilateral burr hole	450	No	Normal	1 day	Left cerebellar
Hyam et al.^26^	79 M	Right	Used aspirin platelet count 77 × 10^9^/L	Right burr hole	Not reported	No	Not reported	First time: 8 days Second: 4 days Third: 6 days	SDH SDH Cerebellar
Dinc et al.^27^	48 F	Left	Normal	Left burr hole	Not reported	Yes	Normal	5 days	Right temporal lobe hemorrhage
Missori et al.^28^	83 M	Bilateral	Not reported	Bilateral burr hole	Not reported	No	Not reported	Immediately	Left parietal lobe
Missori et al.^28^	80 M	Left	Not reported	Left burr hole	Not reported	No	Not reported	5 days	Left frontal lobe
Missori et al.^28^	85 M	Bilateral	Not reported	Left burr hole	Not reported	No	Not reported	3 days	Right temporal lobe
Wang et al.^29^	75 M	Bilateral	Normal	Bilateral burr hole	700	No	Well controlled	2 h	Bilateral temporal lobe
Wang et al.^29^	81 M	Right	Rivaroxaban	Right burr hole	500	No	Normal	1 day	Right occipital lobe
Wang et al.^29^	83 M	Right	Normal	Right burr hole	750	No	Normal	2 days	Right occipital lobe
Patibandla et al.^30^	48 M	Bilateral	Not reported	Left burr hole Right craniotomy	Not reported	No	Hypertension	Immediately	Brain stem Right cerebellar Right thalamus, etc.
Seung et al.^31^	77 M	Bilateral	Normal	Bilateral burr hole	Not reported	Yes	Well controlled	Immediately	Bilateral frontoparietal SAH
Chen et al. ^32^	72 M	Bilateral	Not reported	Bilateral YL‐1 aspiration	Not reported	Yes	Normal	1 day	Brain stem Right basal ganglia, etc.
Cohen‐Gadol^33^	52 M	Left	Not reported	Left two burr hole	620	No	Normal	2 days	Right intraventricular Right SAH
Tyngkan et al.^34^	40 F	Left	Not reported	Left burr hole	Not reported	No	Not reported	2 h	Brain stem
Gader et al.^35^	70 M	Left	Normal	Left two burr hole	Not reported	No	Normal	1 day	Brain stem

Abbreviations: CSDH, chronic subdural hematoma; F, female; M, male; SDH, subdural hematoma.

There are various treatments for CSDH, which include hematoma evacuation through craniotomy or an endoscope, BHS with subdural drainage or subcutaneous drainage, BHS without drainage,^36^, ^37^, ^38^ middle meningeal artery embolization,^39^, ^40^ and steroid or statin therapy.^41^, ^42^ Among them, BHS is the most common surgical procedure; it is simple, minimally invasive, and effective.^43^ We performed BHS with drainage for this patient. In the beginning, we hesitated to operate immediately. The main concerns are as follows: First, after CPR, the impaired consciousness of the patient returned to normal, and there were no obvious neurological symptoms. Second, we believed that the patient was at high risk for surgery, because of his advanced age, a history of atrial fibrillation and cardiac arrest, and a high INR value. Therefore, we decided to wait for BHS until the pacemaker was implanted and the INR recovered to normal. However, on the second day, the patient developed sudden brain hernia, so we had to operate on him immediately. Although the intracranial pressure was relieved after drainage, the patient did not wake up and developed AIH. In conclusion, the timing of surgery is a problem worthy of attention. Although there has been no evidence‐based research about the timing of surgery for elderly patients with CSDH, scholars believe that it may be better for patients with neurological symptoms to receive surgical treatment as soon as possible. Moreover, some studies have shown that preoperative use of anticoagulants is not associated with increased postoperative hematoma.^44^ For instance, Yeon et al.^45^ reported that 15.8% of patients who received warfarin preoperatively and reused warfarin 3 days after surgery had rebleeding, similar to the incidence in the general population.^46^, ^47^ However, other studies suggested that patients taking anticoagulants such as warfarin should not undergo surgery until their INR falls below 1.4 or even 1.2.^48^ Furthermore, Gazzeri et al. reported that use of an anticoagulant before operation was significantly associated with postoperative AIH.^49^ In addition, one case report that we retrieved showed that the patient, who had an INR of 2.8, had subarachnoid hemorrhage after surgery. Similarly, in the present case, it was revealed that during the period when INR was higher than normal, the volume of hematoma tended to increase, while it stopped increasing as the INR recovered to normal. Therefore, we believe that it may be more important to choose the operation time according to the preoperative INR value. It may have been a better option for this patient to undergo surgery as soon as the temporary pacemaker was installed, and the INR value was corrected to the normal level.

Previous studies revealed that the incidence of postoperative AIH was 0.6%–5.1% for patients with CSDH. AIH could occur in various regions such as the brain stem,^50^ cerebellum,^23^ cerebral hemisphere,^51^ ventricle,^52^ subarachnoid space, and bilateral multiple parts.^31^ So far, the mechanism of bleeding is still unclear. Many studies have revealed that bleeding may be attributed to several aspects, such as intracranial hypotension resulting from removal of supratentorial hematoma,^53^ abnormal blood coagulation,^54^ obstruction of jugular venous return caused by excessive head torsion,^55^ injury of venous vessels due to shift of intracranial tissue caused by excessive cerebrospinal fluid drainage,^56^ impaired vascular self‐regulation due to edema of brain tissue compressed by hematoma, decreased response to carbon dioxide, and rupture of vulnerable subarachnoid vessels caused by cerebral hyperperfusion and cortical hyperemia.^57^ It has been proven that the blood volume of brain tissue for patients with CSDH is decreased before an operation^58^ and increased after an operation, and there is also evidence of the displacement of brain tissue during an operation.^59^ In addition, the vascular structure of the elderly is relatively fragile, which cannot tolerate the rapid recovery of normal blood flow. In our case, brain tissues of the patient had been compressed by subdural effusion and hematoma for a long time. Within 30 min, 160 ml of blood fluid was drained from the left subdural and 100 ml from the right subdural. Large changes of intracranial volume in a short period of time lead to large fluctuations of intracranial pressure and pressure imbalance on both sides of the hemisphere, which could result in brain shift, hyperperfusion, and hyperemia. As a result, the patients occurred vascular rupture. In addition, to our knowledge, only one previous study has reported that a high INR value was associated with postoperative hemorrhage. In the current case, we found that the volume of intracranial hemorrhage altered according to dynamic changes of INR. Therefore, we speculate that AIH of current patient may also be related to coagulation abnormality.

How can the risk of postoperative AIH be avoided or reduced for elderly patients with CSDH? Most previous studies suggested slowing down the drainage speed or volume. However, there is no prospective or retrospective study on the safe range of drainage. This paper lists the related case reports (Table 2), in which the minimum drainage volume is 30 ml in 1 h and the maximum drainage volume is 700 ml in 2 h. Therefore, it may be safer to make the drainage volume less than 30 ml in 1 h. This is simple and effective for controlling the drainage speed by raising the drainage bag.^29^ Some scholars believe that it would be helpful if slow drainage of bilateral CSDH is carried out at the same time, and others also confirm that the risk of hemorrhage can be reduced by both drainage with a speed‐limited YL needle and replacement of hematoma with an equal volume of normal saline.^4^, ^59^ However, this patient had cerebral hernia and required rapid decompression, which may not be suitable for the above method. Therefore, for elderly patients as in this case, we believe that it the priority should be to prevent hematoma enlargement early. Also, the following methods are worth considering. First, the patient should better receive head CT or MRI scan regularly and caution should be exercised to avoid falling. Second, if the patient falls, but has no neurological symptoms, some drugs such as steroids and statins can be administered early to stop the following biochemical reaction of CSDH; in addition, embolism of the middle meningeal artery may be useful. Third, if the volume of CSDH does not decrease, after correcting INR, both speed‐limited drainage with YL needle and the replacement of hematoma with saline should be performed as soon as possible to relieve hematoma and prevent cerebral hernia. Finally, if there is a sign of cerebral hernia, surgical treatment should be carried out as soon as possible.

In short, this paper analyzed the causes of postoperative hemorrhage in a patient with subdural effusion, hematoma, and cerebral hernia, discussed the timing of the operation and preventive measures, and reviewed the relevant literature.

## 4 CONCLUSION

The reasons for postoperative AIH are probably associated with fluctuations of intracranial pressure, brain shift, hyperfusion and cortical hyperemia, impaired vascular autoregulation, and coagulation abnormality. To avoid the complication, the speed of bilateral drainage should be slowed using a controllable device, the drained blood should be replaced simultaneously with the same volume of normal saline, and the coagulation abnormality should be corrected before surgery. In the future, after finding subdural effusion, we suggest that these patients take medicine in time and receive surgical drainage or middle meningeal artery embolization, which may help to avoid the above situation.

## CONFLICT OF INTERESTS

The authors declare no conflict of interests.

## ETHICS STATEMENT

Written informed consent was obtained from the patient for publication of this case report.

## AUTHOR CONTRIBUTIONS

Peng Wen was involved in development of the idea, data collection, and writing of the article. Wen‐Long Xu provided technical guidance. Huan Chen was involved in the revision of the article.

## Data Availability

The authors confirm that the data supporting the findings of this study are available within the article.
